# Deciphering the Role of Androgen in the Dermatologic Manifestations of Polycystic Ovary Syndrome Patients: A State-of-the-Art Review

**DOI:** 10.3390/diagnostics14222578

**Published:** 2024-11-16

**Authors:** Ach Taieb, Amri Feryel

**Affiliations:** 1 Faculty of Medicine of Sousse, University of Sousse, Sousse 4000, Tunisia; feryel.amri@gmail.com; 2 Department of Endocrinology, University Hospital of Farhat Hached Sousse, Sousse 4031, Tunisia; 3 Laboratory of Exercise Physiology and Pathophysiology, L.R.19ES09, Sousse 4054, Tunisia; 4 Department of Dermatology, University Hospital of La Rabta, Tunis 1007, Tunisia

**Keywords:** androgens, PCOS, testosterone, skin, hirsutism, acne, female pattern hair loss

## Abstract

Polycystic ovary syndrome is a presvalent endocrine disorder affecting women globally, characterized by hormonal imbalance, metabolic disturbances, and reproductive dysfunction. Diagnosis relies on clinical evaluation, medical history assessment, physical examination, and laboratory tests, with the Rotterdam criteria being widely used. The pathophysiology of PCOS involves genetic predisposition, environmental factors, and lifestyle influences, with hormonal dysregulation, particularly elevated androgens, insulin resistance, and chronic inflammation, playing a pivotal role. These mechanisms not only contribute to reproductive and metabolic disturbances but also to the various cutaneous manifestations, such as acne, hirsutism, and alopecia. This review aims to analyze the role of androgens in the dermatological manifestations in patients with polycystic ovary syndrome, providing insights into underlying mechanisms and guiding the development of effective therapeutic strategies. By synthesizing available evidence, this review aims to deepen understanding of related dermatological manifestations and improve outcomes for affected individuals.

## 1. Introduction

Polycystic ovary syndrome (PCOS) represents one of the most prevalent endocrine disorders affecting women of reproductive age worldwide [1]. Over the past few decades, its prevalence has been steadily rising, posing significant challenges to public health systems globally. PCOS is a complex, multifactorial condition characterized by hormonal imbalance, metabolic disturbances, and reproductive dysfunction [2]. Alongside its well-documented effects on fertility and metabolic health, emerging evidence suggests a profound impact of PCOS on dermatological manifestations, which significantly affect the quality of life of affected individuals.

Diagnosing PCOS involves a combination of clinical evaluation, medical history assessment, physical examination, and laboratory tests. There is no single definitive test for PCOS, and diagnosis requires the exclusion of other conditions that may mimic its symptoms. The diagnostic criteria for PCOS have evolved over time, with various organizations providing different guidelines [3]. However, the used criteria for diagnosis include those established by the 2023 international guidelines criteria, which require the presence of at least two of the following three features (Table 1):

- Menstrual irregularities: these may include oligomenorrhea or amenorrhea.
- Clinical or laboratory evidence of hyperandrogenism: Clinical evidence may manifest as hirsutism, acne, or male pattern baldness. Laboratory evidence involves elevated levels of androgens, such as testosterone, either measured directly or inferred through elevated levels of androgenic hormones like Dehydroepiandrosterone Sulfate (DHEAS).
- Polycystic ovaries on ultrasound examination or enlargement of the ovary’s size or increased anti-Müllerian hormone (AMH) levels [3].

The exact pathophysiology of PCOS remains elusive, but it is widely accepted to involve a combination of genetic predisposition, environmental factors, and lifestyle influences. Hormonal dysregulation, particularly elevated levels of androgens, insulin resistance, and chronic low-grade inflammation, plays a pivotal role in the pathogenesis of PCOS [2]. These underlying mechanisms not only contribute to the characteristic reproductive and metabolic disturbances but also manifest in various cutaneous manifestations, ranging from acne and hirsutism to alopecia and skin tags.

The rising prevalence of PCOS has sparked growing interest in understanding its comprehensive impact on affected individuals, beyond its reproductive and metabolic implications. Among the myriad of symptoms associated with PCOS, dermatological manifestations stand out as particularly distressing, significantly impairing the psychosocial well-being and quality of life of those affected. Skin-related issues such as acne, hirsutism, and alopecia can cause emotional distress, low self-esteem, and social stigmatization, leading to profound psychological consequences [4].

Acne vulgaris, characterized by the formation of comedones, papules, and pustules on the face, chest, and back, affects a significant proportion of women with PCOS due to increased androgen levels and altered sebaceous gland activity [5]. Hirsutism, the excessive growth of terminal hair in a male pattern distribution, results from androgen excess and affects up to 70% of women with PCOS, leading to significant cosmetic concerns and psychological distress [6]. Furthermore, alopecia, particularly female pattern hair loss, is increasingly recognized as a common manifestation of PCOS, further exacerbating the psychosocial burden of the condition [7].

In addition to these visible cutaneous manifestations, PCOS is associated with other dermatological conditions such as acanthosis nigricans, skin tags, and hidradenitis suppurativa, further underscoring the comprehensive impact of this syndrome on skin health and quality of life [8].

The aim of this review is to analyze the role of androgens in the dermatologic manifestations observed in patients with PCOS. Through a comprehensive examination of the existing literature, this study seeks to provide insights into the mechanisms underlying the development of dermatological symptoms such as acne, hirsutism, and alopecia in PCOS patients, with a particular focus on the influence of androgen excess. By synthesizing available evidence, this review aims to contribute to a deeper understanding of the pathophysiology of PCOS-related dermatological manifestations, potentially guiding the development of targeted therapeutic approaches aimed at alleviating these distressing symptoms and improving the quality of life for affected individuals.

## 2. Main Pathophysiological Features of PCOS

**Figure 1:**
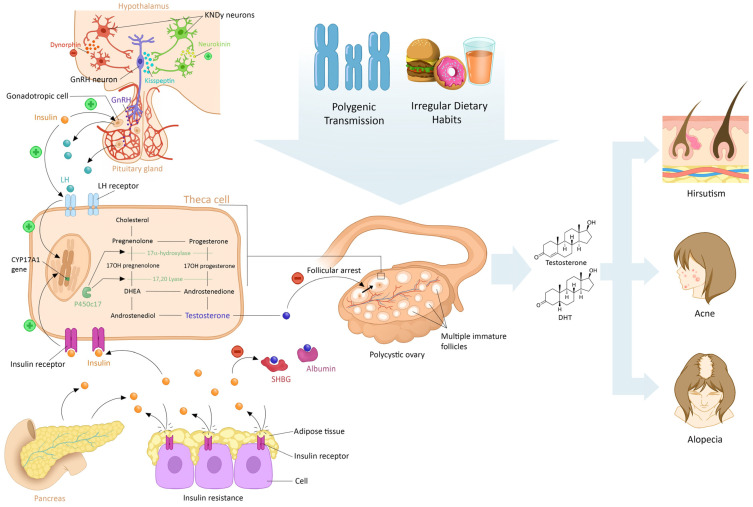
**Main features explaining the pathophysiology of polycystic ovary syndrome (PCOS).** Epigenetic and environmental factors, including dietary habits, contribute to the development of PCOS. Additionally, the dysregulation of the kisspeptin system impacts the pulsatility of Gonadotropin-Releasing Hormone (GnRH), leading to dysfunction in the hypothalamus–hypophyseal–gonadal axis. Particularly in patients with a metabolic phenotype, insulin resistance exacerbates this dysfunction, creating a vicious cycle of excessive androgen production. This cascade of events can manifest in dermatological symptoms such as acne, hirsutism, and female pattern hair loss (FPHL).

The pathophysiology of PCOS is a very complex and heterogeneous phenomenon. Its exact details remain unknown to this day. However, in patients with PCOS, intrication of genetic and environmental factors is an established concept, the effect of both starting at an early age. Herein, we discuss these main pathophysiological elements (Figure 1).

### 2.1. Modifications During Fetal Conception 

During fetal conception, a delicate interplay of genetic and environmental elements shapes a predisposed individual to PCOS. PCOS has been associated with a polygenic nature involving over 241 gene variations, each playing a role in critical functions such as androgen production, ovarian theca cell functioning, insulin resistance, and hypothalamic–pituitary hormone secretion [2,9,10]. Beyond genetic predispositions, epigenetic changes further contribute to PCOS, with these modifications interacting with the gestational environment [11]. Maternal influences during pregnancy, such as hypertension, diabetes, obesity, smoking, stress, and exposure to androgens, drugs, and chemicals, can significantly impact the development of the fetus and increase the likelihood of PCOS manifestation later in life [2]. Notably, the hypothalamic–pituitary–gonadal axis, a key regulator of reproductive function, is programmed by early androgen exposure, emphasizing the critical role of hormonal influences during the formative stages of development [12,13,14]. Understanding the intricate connections between genetic predispositions, epigenetic changes, and the gestational environment provides valuable insights into the complex origins of PCOS. To further underline the importance of a terminology shift, recognizing the multifactorial roots of PCOS, from genetic predispositions to epigenetic modifications and environmental exposures, may enhance understanding and foster more comprehensive diagnostic and therapeutic approaches [10].

### 2.2. Abnormalities in the Hypothalamus–Pituitary–Ovarian Axis 

In PCOS, there is a complex interplay of aberrant hormonal pathways, and one pivotal axis implicated in its pathogenesis is the hypothalamic–pituitary–gonadal axis. The hypothalamus plays a crucial role by secreting gonadotropin-releasing hormone (GnRH) at an increased pulse frequency, a deviation that may be attributed to an inherent defect in the GnRH pulse generator during conception or lower progesterone levels observed in women with PCOS [15,16]. Normally, progesterone acts to slow the pulse frequency of GnRH in healthy individuals [17]. This heightened frequency of GnRH pulsation prompts the anterior pituitary gland to preferentially release luteinizing hormone (LH) over follicle-stimulating hormone (FSH) leading to defective androgen synthesis and the progression of insulin resistance [17,18]. The collaborative action of insulin and LH on theca cells further amplifies the production and secretion of androgens [19,20,21]. Furthermore, at the ovarian level, the intrinsic dysfunction of theca cells contributes to elevated levels of AMH [22].

### 2.3. Consequences of Hyperandrogenism

Hyperandrogenism emerges as a primary trigger in the initiation of PCOS. It plays a pivotal role, not only by giving rise to the clinical manifestations associated with the syndrome but also through the disruption of essential physiological processes [2]. Hyperandrogenism impedes the development of ovarian follicles, leading to the formation of numerous small antral follicles and ultimately resulting in anovulation [20]. Furthermore, in peripheral adipose tissues, high levels of androgen are converted by aromatase enzyme into estrogen, leading to heightened estrogen levels [10]. This multifaceted hormonal imbalance also prompts insulin resistance, exacerbating not just the effects of hyperandrogenism itself but also contributing to dysfunction in both the pituitary and ovaries [23]. The origins of hyperandrogenism within the context of PCOS will be delved into further in Section 3.

### 2.4. Peripheral Insulin Resistance

Peripheral insulin resistance, a hallmark feature of PCOS, is characterized by a dual impairment involving diminished insulin action in response to glucose overload and elevated basal insulin secretion [24]. The origins of insulin resistance in PCOS stem from post-insulin receptor defects, encompassing issues like impaired glucose phosphorylation, reduced glucose oxidation, and disruptions in glucose synthesis and transport [2]. The adipose tissue in women with PCOS contributes significantly to insulin resistance through dysfunctional adipocytokines, decreased adiponectin secretion, perturbations in free fatty acid metabolism, and epigenetic abnormalities affecting the function of glucose transporter 4 [25].

Notably, although insulin resistance is more prevalent in obese women with PCOS, it is essential to highlight that 20% of lean individuals with PCOS also experience insulin resistance, underscoring its potential to exist independently of weight status [26]. This insulin resistance triggers hyperinsulinemia, which culminates in several adverse effects. These include: a decrease in the synthesis of sex hormone-binding globulin (SHBG), consequently elevating free testosterone levels, and also, the stimulation of ovarian theca cells to secrete LH, leading to excess androgen production—a vicious cycle perpetuating the hormonal imbalance in PCOS [21]. Additionally, insulin resistance in PCOS contributes to dyslipidemia, further complicating the metabolic landscape of this syndrome [27].

### 2.5. Effects of Lifestyle on PCOS

Establishing a cause-and-effect relationship between environmental factors and PCOS can be challenging, but certain extensively studied factors in the literature suggest a likely association. **Unhealthy eating habits**, characterized by the consumption of food high in sugars and advanced glycation end products (**AGEs**), stand out as contributors to PCOS. Exogenously consumed AGEs, prevalent in fast food, are found in elevated levels in PCOS patients, leading to cellular damage, modification of steroidogenesis and folliculogenesis, promotion of oxidative stress, and the induction of insulin resistance through inflammatory responses [28]. The resulting **oxidative stress** instigates deoxyribonucleic acid (DNA) methylation and epigenetic changes, silencing tumor suppressor genes and heightening the risk of tumors in women with PCOS [29]. **Physical inactivity and increased body mass index (BMI)** further exacerbate the condition, as obesity fosters insulin resistance, amplifying androgen production. Androgen excess, in turn, contributes to abdominal obesity, perpetuating a detrimental cycle [30]. Additionally, exposure to environmental chemicals, specifically **endocrine disruptors**, disrupts hormonal balance in genetically susceptible individuals [31]. Behavioral management, dietary modifications, and exercise interventions are recognized strategies to manage PCOS. These interventions, including weight loss, have proven effective in breaking the pathophysiological cycle, restoring ovulation, reducing insulin and testosterone levels, and improving fertility outcomes for women with PCOS [32,33,34].

## 3. Origin of Androgen in PCO Syndrome

Androgens are a group of hormones encompassing DHEA, DHEAS, androstenedione, testosterone, and dihydrotestosterone. These are present in decreasing order of concentration in females [35]. While hyperandrogenism can arise from various causes, PCOS stands out as have the most prevalent etiology in women [36]. Within women with PCOS, the excess production of androgens originates from three primary sources: the ovaries, adrenal glands, and peripheral adipose tissue [37].

### 3.1. Ovarian Theca Cells

**Figure 2:**
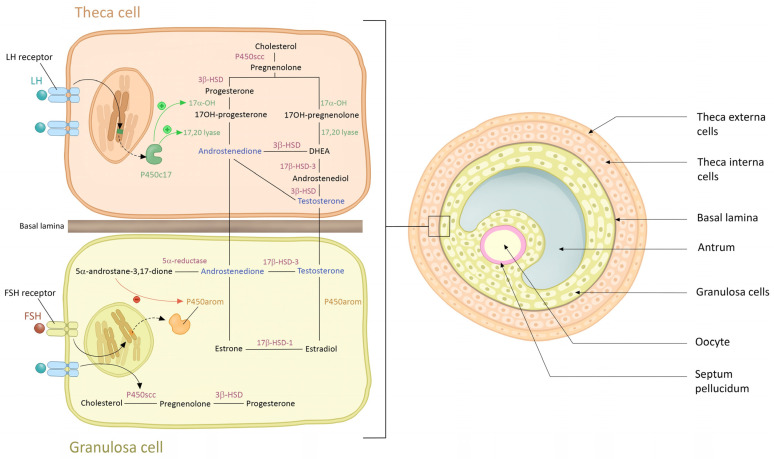
**Biosynthesis of androgens from cholesterol in the ovarian theca cell.** In the ovarian theca cells, cholesterol is converted into androgens, including testosterone and androstenedione, through enzymatic reactions involving cytochrome P450 side-chain cleavage (CYP11A1), 17α-hydroxylase (CYP17A1), and 17β-hydroxysteroid dehydrogenase (HSD17B). In granulosa cells, the conversion of androgens into estrogens involves several enzymatic steps. Testosterone and androstenedione, are transported into the granulosa cells. The enzyme aromatase catalyzes the aromatization of androgens into estrogens, the primary estrogen in females.

Theca cells stimulated by LH biosynthesize androgens from cholesterol, with studies revealing overactivity and overexpression of steroidogenic enzymes in these cells, such as CYP17A1, CYP11A1, HSD3B2, SRD5A1, and 17β-HSD5 (also known as AKR1C3) [38] (Figure 2).

### 3.2. Adrenal Glands

Adrenal glands contribute to androgen production through an imbalance in cortisol steroidogenesis, driven by hypersecretion of adrenocorticotropic hormone (ACTH) in women with PCOS [37]. Androgen biosynthesis occurs in the zona reticularis, which is one of the three layers of the adrenal gland [39].

### 3.3. Adipose Tissue

Particularly in women with obesity, adipose tissue possesses a complete steroidogenic machinery capable of converting circulating androgens to more potent forms. Findings also suggest the presence of key proteins like steroidogenic acute regulatory protein (StAR) and cytochrome P450 family 11 subfamily A member 1 (CYP11A1) in adipose tissues, facilitating de novo steroidogenesis [40,41].

Furthermore, studies propose contributions from hair follicles and genital skin to androgen biosynthesis [42]. Major androgen precursors include DHEAS, DHEA, and androstenedione, while potent androgens like testosterone and dihydrotestosterone are the ones that induce most biological effects by binding to androgen receptors [35]. Metabolized by the liver, these androgens lead to metabolic waste excretion through urine [37].

In women with PCOS, the distribution of androgens is intricately determined by their diverse sources of production within the body. Approximately 50% of DHEA is secreted by the adrenal zona reticularis, with an additional 20% originating from the ovaries. The remaining 30% results from the conversion of circulating DHEAS. Androstenedione, another significant androgen precursor, is synthesized in equal proportions by both the adrenal gland and the ovary [43]. Testosterone, a bioactive form of androgen, is secreted equally from the adrenal zona fasciculata and the ovary. Dihydrotestosterone, a potent androgen, primarily stems from the transformation of testosterone by the enzyme 5α-reductase (5αRD) in peripheral tissues such as the liver, adipose tissue, and the pilosebaceous unit [43]. Additionally, a minor amount of dihydrotestosterone is synthesized by the adrenal zona fasciculata [43]. This intricate network of androgen precursor secretion underscores the dynamic and diverse sources contributing to the androgen excess observed in women with PCOS.

## 4. Physiological Effect of Androgen on Skin and Phaneres in Women

Approximately 66% of testosterone in the bloodstream is bound to SHBG, while 33% is bound to albumin, leaving only 1% in its free, biologically active form [44]. The physiological effects of androgens, particularly their impact on the skin, are primarily mediated by the concentrations of free testosterone and dihydrotestosterone, as the protein-bound fraction remains inactive [44]. Androgenic effects are orchestrated through binding to nuclear receptors within cells [39].

In physiological conditions, androgens exert diverse effects on the skin, with significant implications for hair growth [45]. Androgens play a pivotal role in regulating human hair follicles, particularly during puberty when they stimulate the development of axillary and pubic hair in both sexes. Androgens influence various aspects within the follicle, including the duration of hair growth, the size of the dermal papilla, and the activity of dermal papilla cells, keratinocytes, and melanocytes [46]. However, the response to androgens varies across body sites, leading to the enlargement of hair follicles in androgen-dependent areas while paradoxically fostering miniaturization and a decrease in hair during the anagen stage in scalp follicles [47].

Moreover, androgens contribute to the growth and differentiation of sebaceous glands, with a notable impact on facial sebocytes [48]. Although androgens stimulate sebocyte proliferation, their ability to modify sebocyte differentiation is limited and requires co-incubation with peroxisome proliferator-activated receptor (PPAR) ligands [49].

Additionally, androgens influence epidermal barrier homeostasis and wound healing [50,51]. Testosterone, in particular, has been shown to disrupt epidermal barrier homeostasis and inhibit cutaneous wound healing, enhancing the inflammatory response in adult human skin. Blockade of androgen action through androgen receptor (AR) antagonism accelerates wound healing significantly [50,51]. Beyond skin-related effects, androgens also play a role in coordinating with estrogens to maintain the balance of the female reproductive endocrine system and contribute to increased muscle mass, bone growth, calcium deposition, and red blood cell production [52].

## 5. Hyperandrogenism: Criteria and Diagnostic Methods

Defining hyperandrogenism in PCOS involves both clinical and biochemical considerations, and while they often align, evidence suggests instances of divergence. Clinical hyperandrogenism, encompassing symptoms like hirsutism, acne, seborrhea, androgenic alopecia, acanthosis nigricans, and skin tags, may manifest in women without concurrent biochemical hyperandrogenemia, and vice versa [53,54].

Biochemical hyperandrogenism, also termed hyperandrogenemia, is characterized by elevated serum levels of androgens, including total testosterone, free testosterone, DHEAS, and androstenedione [35]. Challenges in evaluating hyperandrogenism arise from the limited availability and cost of tests, compounded by inconsistent recommendations for testing timing, laboratory variations, and different reference ranges. Androgen levels exhibit variability influenced by factors such as age, menstrual cycle day, and time of day [35,44].

For accurate measurement, it is recommended to obtain the total testosterone level in the early morning during the initial follicular phase of the menstrual cycle, and outside of hormone-modulating therapies [55,56]. In women without cyclical variations, testing can be performed regardless of timing. However, other laboratory procedures generally remain unchanged [35].

To enhance diagnostic precision, mass spectrometry-based assays and calculated measures like the free androgen index (FAI) are employed, with FAI values ≥6 indicative of abnormality [55]. Notably, androstenedione emerges as a more sensitive test for assessing androgen excess compared to testosterone levels in PCOS women [57]. The findings of a study involving 86 PCOS women meeting the Rotterdam criteria revealed that while elevated serum testosterone levels were present in 65% of the participants, serum androstenedione concentrations exceeded the reference range in 88% [58].

Additional tests, including prolactin levels, thyroid function tests, and 17-hydroxyprogesterone, may be required to rule out alternative causes of hyperandrogenemia [35].

Hyperandrogenism is integral to the Rotterdam criteria, serving as one of the three key diagnostic criteria, where the presence of two out of three is necessary for a PCOS diagnosis [3]. This characteristic is prevalent in three out of the four possible PCOS phenotypes, namely A, B, and C, wherein either clinical or biochemical evidence of hyperandrogenism is observed. Notably, hyperandrogenism is absent in phenotype D [1].

In light of a contemporary perspective highlighting the pivotal role of metabolic imbalance in PCOS, hyperandrogenism is redefined as “metabolic hyperandrogenism”. This paradigm shift leads to the categorization of phenotypes characterized by androgen excess as endocrine metabolic syndrome, with or without accompanying ovarian dysfunction [59]. Conversely, the phenotype lacking hyperandrogenism is considered the authentic PCOS, emphasizing ovarian onset over metabolic factors [59]. Consequently, hyperandrogenism assumes a significant role in various aspects of PCOS management. Firstly, it plays a crucial role in confirming the diagnosis, serving as a fundamental criterion. Additionally, it serves as an indicator of metabolic syndrome and the risk of skin manifestations. Moreover, this new understanding of disease etiology allows for better-adapted therapeutic approaches.

## 6. Role of Skin Manifestations in the Diagnosis of PCOs

The recognition of androgens’ role in cutaneous disorders dates back to as early as the 4th century BC when Aristotle observed the correlation between androgenetic alopecia and gender or sexual maturity [60]. In this section, we aim to examine the cutaneous manifestations of hyperandrogenism, delving into each symptom’s definition, clinical evaluation methods, prevalence in patients with PCOS, correlation to androgen values, and the current available therapies and treatment guidelines.

### 6.1. Hirsutism

**Figure 3:**
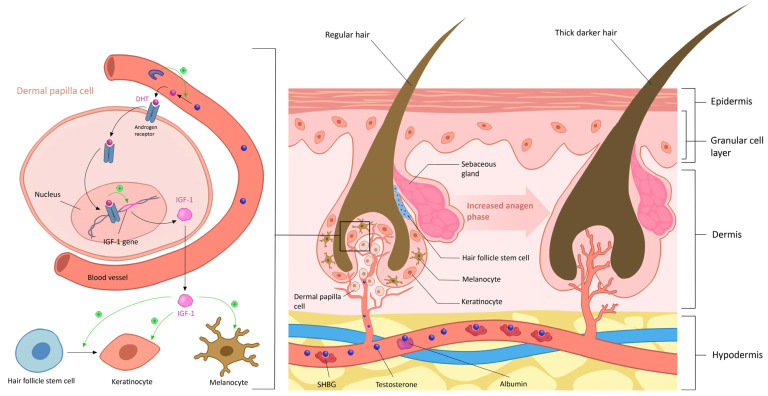
**Targets and effect of androgen in hair follicles leading to conversion of vellus to terminal hair.** Androgens exert their effects on hair follicles primarily through binding to androgen receptors located within the follicular cells. Androgenic hair growth occurs through several mechanisms: **Activation of androgen receptors**: Dihydrotestosterone (DHT) binds to androgen receptors located within the dermal papilla cells of hair follicles. This binding triggers a cascade of intracellular events that promote the growth of terminal hairs. **Prolongation of anagen phase**: Androgens can prolong the duration of the anagen (growth) phase of the hair cycle. This results in increased growth of terminal hairs and a longer lifespan for these hairs compared to vellus hairs. **Stimulation of hair follicle size and thickness**: Androgens influence the size and thickness of hair follicles, leading to the development of larger, more robust terminal hairs compared to vellus hairs. **Induction of hair follicle transformation**: Androgens can induce the transformation of vellus hair follicles into terminal hair follicles. This process involves changes in gene expression and signaling pathways within the follicular cells.

Physiologically, terminal hair in women is predominantly found in areas such as the eyebrows, eyelashes, scalp, axilla, and pubis [37]. Hirsutism is characterized by the presence of such terminal hair in women, additionally following a male pattern distribution [46]. This male sexual pattern manifests in androgen-sensitive anatomical sites, including the face, chest, breast areola, linea alba, lower back, buttocks, and inner thighs [46]. In women with PCOS, the occurrence of hirsutism has an estimated prevalence of 65–75% and emerges as the main manifestation of hyperandrogenism [21]. This symptom is associated not only with androgen excess but also with the individual sensitivity of the pilosebaceous unit to androgens [6] (Figure 3).

#### 6.1.1. Androgen Excess

Androgens play a crucial role in influencing various aspects of hair growth, as evidenced by studies indicating their ability to increase hair follicle size, hair diameter, and the duration of the anagen stage [47]. The acceleration of hair cycles and prolonged anagen phase contribute to the enlargement of follicles, leading to the production of longer and thicker hair in androgen-dependent body areas [47]. While pubic and axillary hair are quite sensitive to small amounts of androgens, other areas may need a higher androgen concentration for follicle terminalization [61]. In areas naturally having vellus hair in women, androgen excess orchestrates, therefore, a transition from vellus to terminal hairs, leading to hirsutism. Notably, women with androgen levels surpassing twice the upper limit of the reference range often experience some degree of hirsutism [62]. However, paradoxes emerge such as the poor correlation of the severity of hirsutism with the measured androgen levels using conventional techniques and the existence of idiopathic hirsutism (in women with excessive body hair growth despite normal plasma androgen levels) [62]. Possible explanations for these incongruities include relative hyperandrogenemia at the tissue level resulting from a lower estradiol/testosterone ratio in these individuals, and the existence of individual variability in terms of follicles’ sensitivity to androgens [63]. Therefore, it becomes evident that androgen measurements should not serve as a substitute for clinical judgment.

#### 6.1.2. Sensitivity of Hair Follicles to Androgens

Hair follicles, which broadly express androgen receptors, also house androgen-metabolizing enzymes such as cytochrome P450 aromatase, type 2 17β-hydroxysteroid dehydrogenase, and 5αRD. These enzymes are critical for regulating androgen levels within the follicle [6,64,65]. An alteration in their expression may be linked to varying androgenic activity levels [65]. Additionally, microRNAs (micro ribonucleic acids) have been identified as contributors to the regulation of hair follicle morphogenesis and regeneration, yet results regarding their exact effects on receptor activity remain contradictory [66]. Despite this knowledge, numerous questions persist regarding the precise mechanisms and the signaling pathways that govern androgen/AR interaction.

Currently, the **modified Ferriman–Gallwey (mFG)** scoring system is the most employed tool in clinical settings to visually assess excessive terminal hair, offering a standardized approach to evaluate hirsutism and enabling data comparison. This scoring system evaluates hair growth in nine body areas: the upper lip, chin, chest, upper and lower back, upper and lower abdomen, upper arm, and thigh. The mFG assigns scores from 0 (no terminal hair) to 4 (male pattern hair) in each area, with intermediate scores (1, 2, and 3) indicating varying levels of body hair growth, yielding a maximum score of 36 [61,67].

In areas where hair is shaved, self-scoring proves to be clinically useful, particularly for follow-up assessments. However, clinicians should keep in mind its modest correlation with scores determined by a trained observer [68]. A cutoff value based on a predominantly Caucasian cohort defines hirsutism with a total score of 8 or higher [67]. And while a universal mFG score cutoff would be beneficial for cross-population comparisons, it is essential to consider substantial variability elements such as: ethnic origin, skin type, age, and associated comorbidities like obesity and insulin resistance [69]. In fact, the 2018 international evidence-based guideline for PCOS management suggests factoring in ethnic origin when interpreting mFG scores across populations [69]. Ethnic-specific mFG score cutoffs, recommended by the Endocrine Society, are available. For instance, the suggested cutoff is ≥8 for black or white women in the United States and the United Kingdom, ≥9 to ≥10 for Mediterranean, Hispanic, and Middle Eastern women, ≥6 for South American women, and a range of ≥2 for Han Chinese women to ≥7 for Southern Chinese women [67,70].

In studies examining the correlation between mFG scores and skin type, findings indicate that patients with skin types V and VI exhibit the highest mFG scores and hirsutism prevalence, followed by skin types III and IV, and lastly, by skin types I and II [70]. Age is an additional consideration in hirsutism evaluation, as hyperandrogenism may partially resolve before menopause in women with PCOS [71]. To ensure sensitivity in score interpretation, it is imperative to consider all these factors collectively.

### 6.2. Female Pattern Hair Loss (FPHL) 

Female pattern hair loss (FPHL) is a condition encompassing various disorders characterized by hair loss following the typical pattern of female androgenetic alopecia. The distinctive features of FPHL involve a gradual and diffuse thinning of hair across the central scalp, while the frontal hairline typically remains intact [72]. The perceived thinning of hair results from a reduction in hair density and the miniaturization of hair follicles, attributed to the shortening of the anagen phase, which increases the conversion of terminal hairs to vellus-like hairs. It is usually reported by patients as increased visibility of the scalp through the hair [35].

This form of non-scarring alopecia becomes more prevalent in women as they age. The diagnosis is often clinical, established based on observed symptoms, with trichoscopy serving as a potential auxiliary tool. However, differentiating FPHL from other forms of non-scarring alopecia, such as telogen effluvium, thyroid abnormalities, iron deficiency, and alopecia areata, can pose diagnostic challenges [72]. FPHL was found to have a prevalence of 22% in subjects meeting diagnostic criteria for PCOS [73].

In contrast to male androgenetic alopecia, FPHL does not lead to complete baldness [74]. Despite this, the condition can exert a significant psychological toll on individuals, contributing to heightened levels of anxiety and depression [75].

#### 6.2.1. Role of Androgens in Female Pattern Hair Loss

The primary factors contributing to hair loss in PCOS include polygenic susceptibility and heightened androgen action within the scalp, potentially compounded by chronic low-grade inflammation [7,35,72].

- Androgen Excess

**Figure 4:**
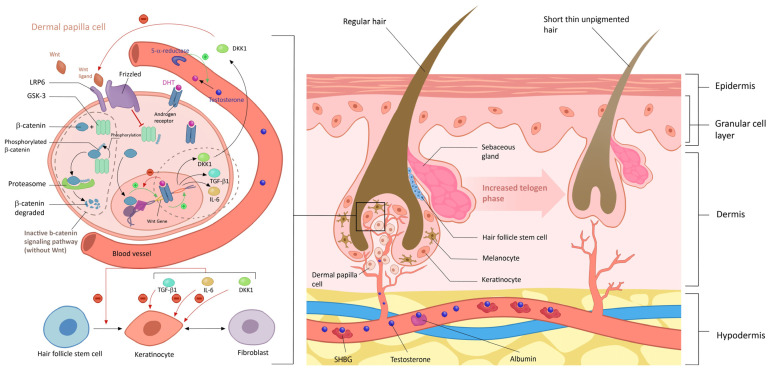
**Paradoxical effect of dihydrotestosterone on the scalp leading to miniaturization of hair follicle.** A focus on the role of 5α-reductase, an enzyme that converts testosterone to dihydrotestosterone (DHT), which contributes to the shrinking of hair follicles, and shortening of the anagen phase leading to thinner and weaker hair strands on the scalp. The mechanisms underlying DHT-induced miniaturization of hair follicles include **shortening of anagen phase**: DHT can shorten the duration of the anagen (growth) phase of the hair cycle, leading to reduced hair growth and shorter hair length; **decreased hair follicle size**: DHT causes a reduction in the size and diameter of hair follicles, resulting in the production of thinner, weaker hairs; **increased telogen phase**: DHT prolongs the telogen (resting) phase of the hair cycle, leading to delayed shedding of miniaturized hairs and a decrease in the overall density of hair on the scalp; and **disruption of hair follicle cycling**: DHT interferes with the normal cycling of hair follicles, disrupting the balance between hair growth and hair shedding.

In an intriguing paradox, DHT, typically known for promoting hair growth and development in various body regions, exhibits a contrasting effect on the scalp, leading to the transformation of terminal hairs into vellus hairs through an effect on the dermal papillae. This unique observation underscores the intricate regulatory mechanisms governing hair follicles across different anatomical regions [7] (Figure 4).

The prevailing belief in the androgen-mediated nature of FPHL is supported by multiple factors. Firstly, the established relationship between DHT and male androgenetic alopecia, which provides a foundational understanding of androgenic influences on hair loss [64]. Additionally, women with hyperandrogenic conditions, such as PCOS and congenital adrenal hyperplasia, frequently experience early-onset FPHL, emphasizing the connection between androgen imbalance and female hair loss [74]. The impact of hormonal shifts during menopause, characterized by increased androgen and decreased estrogen levels, further contributes to these effects on hair, including diminished diameter, growth rate, and the percentage of time spent in the anagen phase [76].

However, the relationship between androgens and hair loss exhibits nuances. Observations reveal that FPHL in PCOS is more closely associated with clinical hyperandrogenism rather than biochemical hyperandrogenism. In a detailed case series involving 109 women experiencing moderate to severe FPHL, only one-third exhibited laboratory evidence for hyperandrogenism [74]. An additional layer of complexity is introduced through the postulation that an increased peripheral responsiveness to androgens exists and is genetically determined [7,45].

- Genetic Susceptibility

Extensive studies have supported the intricate relationship between FPHL, local androgen concentrations, and androgen receptor sensitivity, revealing higher rates of local testosterone and DHT production by the 5αRD isoenzyme type II in alopecia-prone areas of susceptible individuals [45]. Genetic variations in androgen sensitivity play a crucial role, specifically through the androgen-mediated inhibition of the Wnt/b-catenin pathway. This pathway holds significance in initiating and maintaining the anagen phase of hair growth [77]. Moreover, excessive tissue-active androgens have been shown to induce apoptosis of dermal papilla cells via the bcl-2 pathway [78]. In addition, recent genetic research has identified associations between sequence variations in the AR gene and estrogen receptor 2 gene (ESR2) and susceptibility to FPHL [78]. These discoveries highlight the intricate genetic factors that influence an individual’s predisposition to this specific type of hair loss.

- Scalp Low-Grade Inflammation

Cases of FPHL were reported in individuals lacking androgen receptors, indicating the potential involvement of an androgen-independent mechanism [79]. In this context, scalp low-grade inflammation emerges as a noteworthy factor. Multiple pieces of evidence suggest its contribution to hair loss [80]. These findings encompass the correlation between an elevated level of prostaglandin D2 (PG-D2) and the miniaturization of hair follicles, as well as the inhibitory effect on hair growth observed with the topical application of PG-D2 [81]. Histological examination of the miniaturization process also reveals an association with a microinflammatory lymphocytic infiltrate in the peri-infundibular region, providing microscopic insights into the pathological changes occurring in FPHL [81].

#### 6.2.2. Clinical Evaluation and Scoring

Three primary patterns of FPHL have been identified. The most prevalent is the “Christmas tree” pattern (Olsen pattern), attributed to frontal hair thinning [82]. Following closely is the second most common pattern known as the Ludwig pattern, characterized by central scalp involvement while sparing the frontal hairline [83]. The third pattern, recognized as the Hamilton pattern, is observed in patients with significant androgenization, manifesting as bitemporal recession and, in rare instances, associated with vertex thinning [72].

**Figure 5:**
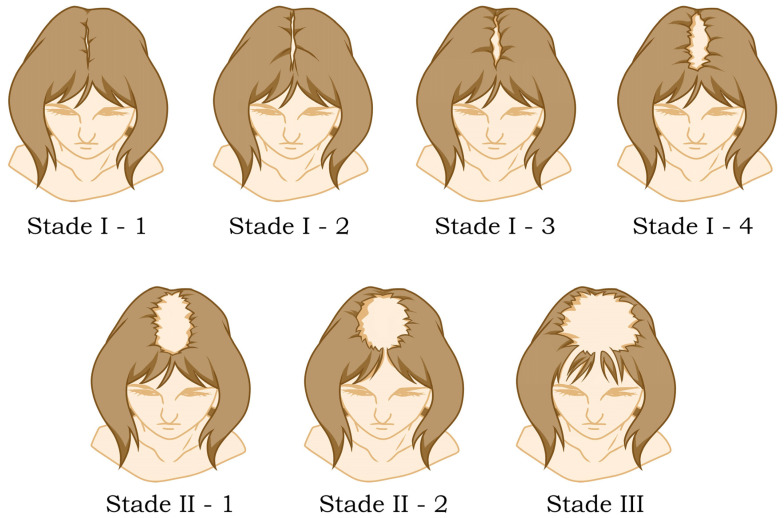
**Ludwig’s scale: A visual tool for assessing the severity of female pattern hair loss (FPHL).** This image presents Ludwig’s Scale, which categorizes hair thinning into three distinct stages: from mild in the first degree to complete absence of hair in the central scalp in the third degree.

To assess hair thinning effectively, a recommended method involves parting the hair vertically in both the central and occipital scalp and comparing these two areas [82]. This approach is particularly useful as the occipital scalp is not under androgen control. The severity of FPHL is often evaluated using Ludwig’s scale, which classifies degrees of thinning from mild in the first degree to complete absence of hair in the central scalp in the third degree [83] (Figure 5).

Alternative scales used for assessment include Savin’s scale (eight stages), Sinclair’s classification, and Olsen’s classification [84].

The pull test, indicative of telogen roots, may be positive in the central scalp during initial phases but is generally negative in long-standing forms. It is essential to note that hair shedding does not uniformly involve the entire scalp, with the possibility of a negative result in the occipital scalp [72]. Trichoscopy, utilizing magnification ranging from 20× to 70×, facilitates the in vivo visualization of the scalp epidermis, follicles, hair shafts, and vascular patterns in FPHL [85]. A key trichoscopical sign in FPHL is the presence of hair diameter variability exceeding 20%, attributed to the miniaturization of some hairs. Additionally, trichoscopy may reveal yellow dots indicating empty follicles, small areas of focal atrichia, and peripilar hyperpigmentation [72,85].

In 2009, Rakowska et al. introduced major and minor dermoscopic criteria for diagnosing FPHL, where the diagnosis is confirmed with the presence of either two major criteria or one major and two minor criteria, as outlined in Table 2 [85].

### 6.3. Acne and Seborrhea 

Acne is an inflammatory condition mediated by the immune system that commonly affects both adolescents and adults, with its prevalence decreasing after the age of 30 and being notably rare during the menopausal period [86]. As an independent factor, acne may contribute significantly to profound psychological discomfort [87]. The intricate and multifaceted pathogenesis of acne encompasses four pivotal factors as follows [88]:

1. Is sebaceous gland hyperplasia leading to excessive sebum production?
2. Irregularities in follicular growth and differentiation resulting in hyperkeratinization and obstruction of the pilosebaceous unit.
3. Cutaneous dysbiosis, characterized by the selection of virulent subtypes of Cutibacterium acnes (*C. acnes*) and/or other bacterial agents.
4. Inflammation and the activation of the innate immune response.

These processes are intricately interconnected and influenced by various hormonal and genetic factors, adding to the complexity of acne’s pathophysiology [88].

A recent meta-analysis explored the prevalence of acne in adult women with and without PCOS. The study, encompassing data from 31 studies with 23,426 women with PCOS and 1,896,979 healthy controls, revealed a 42% prevalence of acne among adult women with PCOS, compared to 17% in the control group [5]. This higher prevalence in PCOS underscores a link to androgen excess and androgen receptors’ sensitivity.

#### 6.3.1. Androgen Excess

The well-established correlation between androgen excess and the development of acne has been extensively documented [35].Various arguments highlight this association. For instance, the onset of acne often coincides with puberty, a period marked by rising androgen levels. Patients with conditions linked to androgen excess, such as PCOS and congenital adrenal hyperplasia (CAH), exhibit higher rates of acne [35]. Notably, areas prone to acne demonstrate an elevated expression of androgen receptors and increased 5αRD activity [89]. Recent findings reveal that 11-oxo androgens of adrenal origin are quantitatively greater in circulation than the levels of typically assessed androgens. However, data on oxy-androgen levels in adult female acne are still currently lacking [90].

Androgens promote sebum production and alter its lipid profile [35]. This excessive sebum serves as a nutrient source for the skin colonizer *C. acnes* [91]. Furthermore, lipases produced by *C. acnes* hydrolyze sebum into pro-inflammatory free fatty acids. These alterations, combined with suppressive effects on neutrophil phagocytosis and reactive oxygen species (ROS) generation, along with a decrease in epidermal barrier function leading to the permeation of chemotactic molecules, contribute to the inflammatory response of the host. This response ultimately causes follicular wall rupture and the formation of inflammatory lesions [92]. Interestingly, up to 60% of adult female patients with acne and normal androgen levels exhibit increased androsterone glucuronide levels, which is a metabolite derived largely from androstenedione, suggesting heightened androgen sensitivity [93].

#### 6.3.2. Receptor Sensitivity

In certain studies, there is a lack of correlation between serum androgen levels and the presence or severity of acne [94]. In a comprehensive study involving 835 post-adolescent females with acne, 43.47% of patients exhibited normal androgen profiles, challenging the idea that androgens, as individual compounds, can significantly influence sebocyte differentiation [95]. Consequently, the formation of acne appears to be more reliant on local androgen concentrations and the sensitivity of sebocytes to androgens [35].

Interestingly, the skin expresses all the necessary enzymes for the conversion of DHEAS to DHT [96]. Aromatase, present in sebaceous glands and the outer root sheath of hair follicles, can modify local androgen action through aromatization [96]. The androgen metabolism within the pilosebaceous unit undergoes further modification by several other steroid enzymes, including sulfotransferase, 3β-hydroxysteroid dehydrogenase, and 17β-hydroxysteroid dehydrogenase [97].

Sebaceous glands possess, additionally, receptors for various growth factors, such as epidermal growth factor, insulin-like growth factor I, and keratinocyte growth factor, all of which play a role in modifying sebum production [96]. These intricate processes highlight the multifaceted nature of androgen influence on acne development within the skin’s structures.

#### 6.3.3. Clinical Evaluation/Scoring

Acne can manifest clinically through both non-inflammatory, such as closed and open comedones, and inflammatory lesions, including papules, pustules, and nodules [88]. These manifestations typically occur on the face, chest, and back, areas with a higher concentration of pilosebaceous glands [8].

The correlation between acne location and a woman’s androgen status remains inconclusive, as recent studies comparing facial acne distribution in women with and without PCOS revealed no statistically significant differences in inflammatory acne distribution [8]. However, specific acne characteristics, such as sudden onset, association with hirsutism or irregular menses, or resistance to conventional treatments, may indicate an underlying androgen abnormality in women [56].

Despite the absence of a unanimous consensus on the preferred acne grading scale, many clinicians commonly use a simplified subjective scale categorizing acne as mild, moderate, or severe. However, a more recommended approach involves semi-objective grading tools, such as counting acne lesions and evaluating severity through comparisons with photographs or figures [88].

The actual recommended acne grading tool is the **Global Evaluation Acne grading scale** [98] (Table 3). It is a widely endorsed tool that assesses acne severity on a scale from 0 (no acne) to 5 (severe acne with numerous inflammatory lesions and nodules across the face). For patients with low scores (scores 0–3), further careful examination of the mandibular region is also recommended for better scoring [99]. A possible alternative grading tool is the Burke and Cunliffe counting method [100].

## 7. Future Perspectives

In contemplating the future perspectives of PCOS, a crucial shift is needed to address the paradox inherent in its definition. It becomes imperative to emphasize etiologic factors over symptomatic manifestations, paving the way for a more nuanced understanding. An exploration into tailored treatments based on phenotypic variations emerges as a promising avenue, steering away from a one-size-fits-all approach.

The high heritability of PCOS prompts a focus on elucidating the underlying mechanisms and strategically targeting the risk of transmission through the activation of epigenetic elements, particularly in symptomatic women during preconception care. Efforts should also be directed toward unraveling the post-birth factors triggering the progression of PCOS, and maybe identifying biomarkers to detect high-risk individuals early in life. 

In regards to significant overlap in hormonal values between women with PCOS and controls, advancements in creating high-quality assays which are accessible seem necessary.

In vitro studies, such as using keratinocyte cultures or sebocyte cell lines, can help elucidate the molecular pathways that link androgens to skin manifestations, particularly acne. However, these models have limitations in replicating the full complexity of PCOS. On the other hand, animal models of PCOS, such as those induced by androgen administration or PCOS-like models in rodents, have been used to study various aspects of the disease, including hirsutism and skin inflammation. These models allow for a better understanding of the mechanistic pathways and the effects of potential therapies. Though more work is needed to optimize these models for dermatological manifestations, they offer valuable insights into how PCOS affects the skin at a biological level.

## 8. Conclusions

In conclusion, the multifaceted nature of PCOS encompasses not only reproductive and metabolic disturbances but also significant dermatological manifestations. Understanding the dermatological pathophysiology of PCOS is paramount for developing more effective treatment targets and improving outcomes for affected individuals. Dermatological manifestations such as acne, hirsutism, and alopecia not only cause physical discomfort but also have profound psychological implications, significantly impacting the quality of life and psychosocial well-being of those with PCOS. By delving into the mechanisms underlying these dermatological symptoms, particularly the role of androgens, researchers and clinicians can identify specific pathways for intervention, leading to more targeted and personalized therapeutic approaches.

Moreover, recognizing the intricate relationship between hormonal dysregulation and dermatological manifestations in PCOS opens doors to novel treatment modalities that address both reproductive and cutaneous symptoms simultaneously. Targeting androgen excess, insulin resistance, and chronic inflammation through pharmacological agents or lifestyle modifications could offer promising avenues for alleviating distressing dermatological symptoms and improving overall patient outcomes.

## Figures and Tables

**Table 1:** **PCOS phenotypes through the evolution of diagnostic criteria.** This table delineates the primary features associated with each PCOS phenotype (A, B, C, and D) and provides a comparative overview of the diagnostic criteria across major guidelines, including NIH 1990, Rotterdam 2003, AE-PCOS Society, and the most recent 2018/2023 updates. The table highlights the presence or absence of key diagnostic features, such as hyperandrogenism/hirsutism, ovulatory dysfunction, and polycystic ovarian morphology (including anti-Müllerian hormone [AMH] levels), as they relate to each phenotype. The evolution in diagnostic criteria demonstrates a trend toward broader inclusion of phenotypic variations, which enhances the identification and classification of PCOS subtypes.

		PCOS Phenotypes			
		A	B	C	D
Features					
	Hyperandrogenia/hirsutism	X	X	X	
	Ovulatory dysfunction	X	X		X
	Echographic polycystic ovaries/AMH	X		X	X
Diagnostic Criteria					
	NIH 1990	X	X		
	AE-PCOS Society	X	X	X	
	Rotterdam 2003	X	X	X	X
	2018/2023 guidelines	X	X	X	X

**Table 2:** **Dermoscopic criteria for diagnosing female pattern hair loss (FPHL) by Rakowska et al.** [85]. The diagnosis is confirmed with the presence of either two major criteria or one major and two minor criteria. It enhances the understanding and accurate identification of FPHL through dermoscopic examination.

Major Criteria	Minor Criteria
(1) More than 4 yellow dots in 4 images in the frontal area. (2) Lower average hair thickness in the frontal area compared with the occipital area. (3) >10% of thin hairs (<0.03 mm) in the frontal area.	(1) Increased frontal to occipital ratio of single-hair pilosebaceous units. (2) Vellus hairs. (3) Perifollicular discoloration.

**Table 3:** **Global Evaluation Acne grading scale,** categorizing acne conditions into six grades based on the type and extent of lesions. This grading system aids in assessing and communicating the severity of acne vulgaris (122).

Grade	Value	Definition
**Clear**	0	Normal, clear skin with no evidence of acne vulgaris
**Almost clear**	1	Rare non-inflammatory lesions present, with rare non-inflamed papules (papules must be resolving and may be hyperpigmented, though not pink-red)
**Mild**	2	Some non-inflammatory lesions are present, with few inflammatory lesions (papules, pustules only, no nodulocystic lesions)
**Moderate**	3	Non-inflammatory lesions predominate, with multiple inflammatory lesions evident; several to many comedones and papules/pustules; there may or may not be one small nodulocystic lesion
**Severe**	4	Inflammatory lesions are more apparent; many comedones and papules/pustules; there may or may not be a few nodulocystic lesions
**Very severe**	5	Highly inflammatory lesions predominate; variable number of comedones; many papules/pustules, and many nodulocystic lesions

## Data Availability

All the scientific data are available on request.
